# Buffalo Medical and Surgical Association

**Published:** 1884-01

**Authors:** Charles Cary

**Affiliations:** Acting Secretary


### Buffalo Medical and Surgical Association.

*Regular Meeting, December 4th, 1883.*

Present—Dr. Cronyn in the Chair, Drs. Mann, Tremaine, Brecht, Coakley, Hinkel, Campbell, Greene, Wyckoff, Bartlett, Cary, Frederick, Clark and Packwood. Drs. Hawkins and Park were present by invitation.

Dr. Howe presented a case of very obstinate conjunctivitis which had yielded to treatment by jequiriti. At the time treatment was begun the cornea was opaque. Result excellent.

Dr. Hartwig read a paper on sponge-tents and their uses in medicine. The article appears in this number.

*Discussion*—Dr. Mann said he regretted being obliged to differ with the essayist in the general use of sponge-tents as suggested by him, owing to the putrefactive changes which these tents produce and the resulting septicæmia. He uses as explorative measures steel instruments and then the tupelo-tent, and only dilates once, and does not use even the tupelo for consecutive dilatation. He would limit the use of the sponge to treatment of chronic enlargement of the uterus where the irritation assisted in bringing about a case.

Dr. Park spoke of the use of slippery elm and rawhide tents, especially in connection with surgery.

Dr. Bartlett spoke of the use of tents in epistaxis, and said he had never seen any bad results follow its use; he also claimed originality in their use.

Dr. Greene spoke briefly on the same subject.

Dr. Cronyn stated that Dr. Bartlett was not the originator of the use of sponge-tents in nasal hemorrhage, but claimed he had suggested it himself to Dr. Bartlett.

Dr. Bartlett replied that he had used it before he had had the pleasure of knowing Dr. Cronyn.

Dr. Hartwig closed the discussion by stating that his paper was to show as well when not to use the tent as when to resort to its use.

*Voluntary Communications*—Dr. Tremaine showed a stone which he had removed from a child three and one-half years old, by supra-pubic operation. He said it was the largest ever removed from a child of that age.

Dr. Park said that the supra-pubic operation was not often performed in this country, but was a familiar operation on the Continent; also that the anatomy of the child seemed especially to recommend this form of operation. He also exhibited a stone which he had removed recently by lithotrity. Weight, 1,290 grs., dry.

Dr. Clark gave the result of a post mortem examination, where he found a fœtus in the abdominal cavity, the result of a ruptured ovarian pregnancy at five months; showed the uterus and appendages.

Moved and carried that the privileges of the association be extended to Drs. Park, Hawkins and Campbell, until such time as they can join.

*Prevailing Diseases*—Scarlet fever, some typhoid fever, measles and dysentery.

Adjourned.

CHARLES CARY,
*Acting Secretary.*

